# miR-191 promotes tumorigenesis of human colorectal cancer through targeting C/EBPβ

**DOI:** 10.18632/oncotarget.2864

**Published:** 2014-12-27

**Authors:** Xiao-Fei Zhang, Ke-ke Li, Lu Gao, Shang-Ze Li, Ke Chen, Jun-Bin Zhang, Di Wang, Rong-Fu Tu, Jin-Xiang Zhang, Kai-Xiong Tao, Guobin Wang, Xiao-Dong Zhang

**Affiliations:** ^1^ College of Life Sciences, Wuhan University, Wuhan 430072, PR China; ^2^ Department of General Surgery, Union Hospital, Tongji Medical College, Huazhong University of Science and Technology, Wuhan 430000, PR China; ^3^ Institute of Cardiovascular Disease, Union Hospital, Tongji Medical College, Huazhong University of Science and Technology, Wuhan 430000, PR China; ^4^ Institute of Urology, Tongji Hospital, Tongji Medical College, Huazhong University of Science and Technology, Wuhan 430000, PR China; ^5^ Department of Emergency, Union Hospital, Tongji Medical College, Huazhong University of Science and Technology, Wuhan 430000, PR China; ^6^ Department of Gastrointestinal Surgery, Union Hospital, Tongji Medical College, Huazhong University of Science and Technology, Wuhan 430000, PR China

**Keywords:** microRNA-191, colorectal cancer, apoptosis, C/EBPβ, tumorigenesis

## Abstract

MicroRNA-191 (miR-191), a small non-coding RNA, is involved in disease development and cancer diagnosis and prognosis. However, how miR-191 functions in colorectal cancer remains largely unclear. In this study, we show that miR-191 is highly expressed in colon tumor tissues, and that inhibition of miR-191 leads to decreased cell growth, proliferation and tumorigenicity in a xenograft model. Overexpression of miR-191 in colorectal cancer cell lines alters cell cycle progression and cell resistance to 5-Fu induced cell apoptosis. Mechanistic studies demonstrated that miR-191 directly binds to the 3′UTR of the C/EBPβ mRNA and mediates a decrease in the mRNA and protein expression of C/EBPβ. We further showed that C/EBPβ induces growth arrest in a colorectal cancer cell line and that its expression is negatively correlated with the miR-191 level in patient samples. Our findings suggest that miR-191 may be a potential gene therapy target for the treatment of colorectal cancer.

## INTRODUCTION

MicroRNAs (miRNAs) are small noncoding RNAs (18~22 nucleotides) that play important regulatory roles in plants and animals by repressing the translation of proteins from mRNAs (messenger RNAs) or by increasing the degradation of mRNAs through binding to their 3′ untranslated regions (3′ UTRs) [1]. Each miRNA regulates the expression of tens or hundreds of proteins and plays critical roles in most biological processes such as cell proliferation, cell survival and inflammatory responses [2, 3]. Aberrant miRNA expression has been frequently found in many cancers and multiple miRNA-expression profiles of human tumors uncovered the correlation between miRNA expression patterns and the tumor type and stage [4, 5]. Depending on the mRNA targets that they regulate, miRNAs can act as oncogenes (e.g., miR-17-92) or as tumor suppressor genes (e.g., miR-15a/16-1, let-7 and miR-34) [6–9].

Colorectal cancer (CRC) is the second leading cause of cancer death for both males and females in the United States [10]. Increasing evidence indicates that deregulation of miRNAs affects cell growth and development of colorectal cancer [11–13]. Specific miRNAs could serve as useful clinical biomarkers and potential therapeutic targets for colorectal carcinoma [14, 15]. Previous work has demonstrated that miR-191 was deregulated in a wide range of human cancers, including breast cancer [16], hepaotocellular carcinoma [17], thyroid follicular tumors [18] and acute myeloid leukemia [19]; and this deregulation may be associated with clinical stage, patient survival and disease prognosis. Interestingly, miR-191 exerts diverse and often conflict biological effects, which are always cell-type and context specific. In hepatocellular carcinoma, miR-191 was identified as an oncogene and its inhibition led to decreased cell proliferation and induced apoptosis *in vitro* and *in vivo* [17]. miR-191 functions as an estrogen inducible oncomiR in breast cancer, and mediate enhanced cell proliferation and migration by targeting SATB1 [20]. In contrast, Di Leva G et al. reported that activation of the miR-191/425 cluster reduced proliferation and impaired tumorigenesis in breast cancer cells [21]. In addition, miR-191 reduced growth and cell migration by targeting CDK6 in thyroid follicular tumor [18]. Several studies have provided strong evidence that miR-191 is overexpressed in human CRC [22–24]. Recently, Dong et al. reported that high miR-191 expression was associated with CRC tumor invasion by directly targeting tissue inhibitor of metalloprotease 3 (TIMP3), a pro-apoptotic gene in various cancers and diseases [25]. However, the molecular mechanism by which miR-191 functions in CRC remains largely unknown. Therefore, identification of the effects of miR-191 and its targets in CRC may lead to new perspectives for gene therapy clinical trials.

In the current study, we examined the expression of miR-191 in different human colorectal cancer cell lines and tissues. We showed that miR-191 expression was significantly up-regulated in colon cancer tissues compared to adjacent non-cancerous lung tissues. Sustained miR-191 overexpression was associated with increased viability, cell proliferation and tumorigenicity *in vivo*, and we further revealed that miR-191 promoted cell resistance to chemotherapeutic agents such as 5-Fu. Mechanistically, we found that miR-191 functioned as an ‘oncomiR’ by directly targeting the tumor suppressor C/EBPβ and that there was a negative correlation between miR-191 and C/EBPβ expression. In addition, C/EBPβ overexpression partially abolished the effects of miR-191 in CRC cells.

## RESULTS

### miR-191 is upregulated in colon cancers

**Figure 1:**
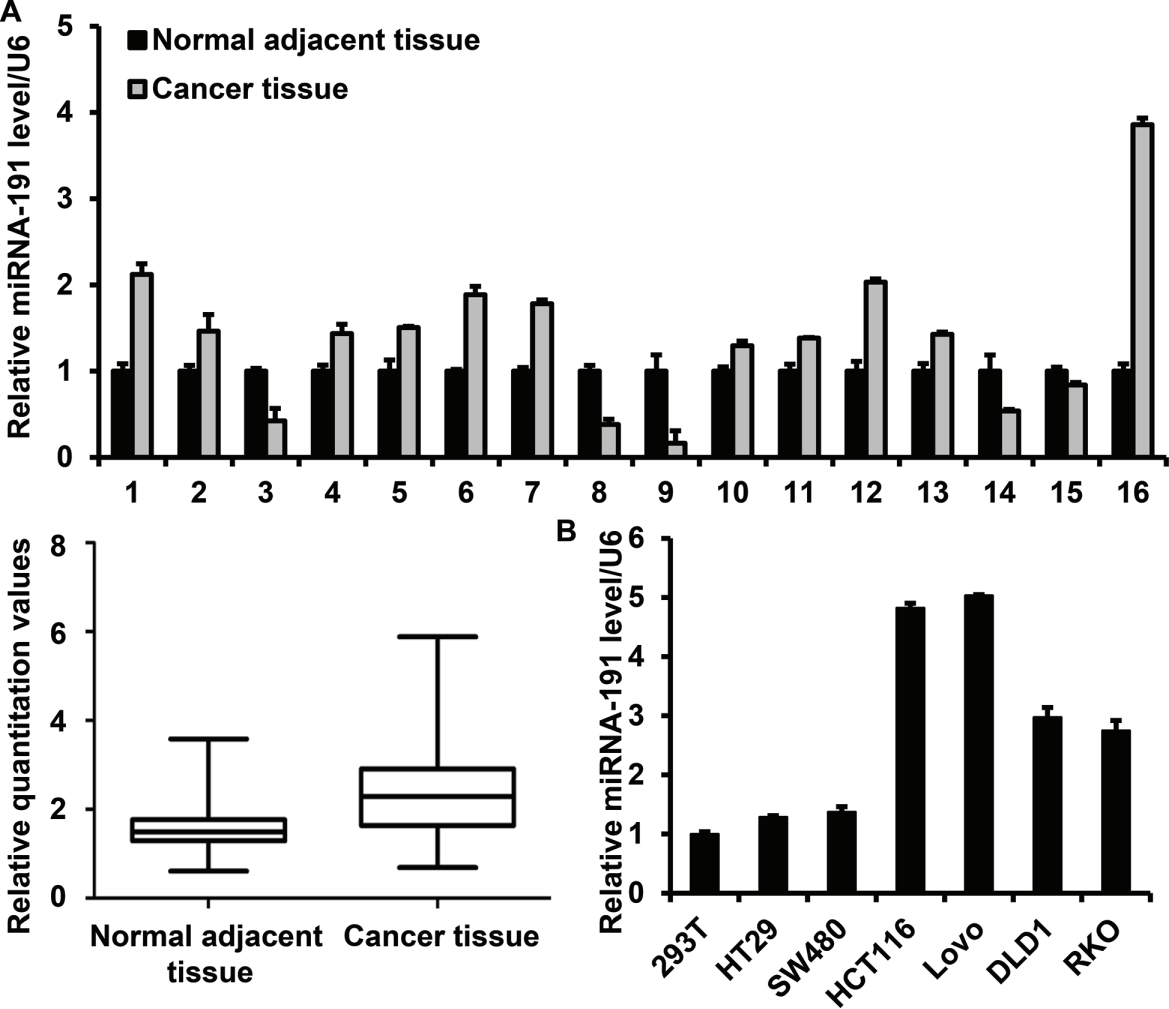
miR-191 is up-regulated in colon cancers Stem-loop RT-PCR analysis of the miR-191 level in tissues and cell lines. **(A)** Relative miR-191 expression in 16 paired colon cancer tissues and adjacent non-tumor tissues (upper panel). miR-191 expression values were expressed as ratios with U6 snRNA (×10). (lower panel, *P* = 0.011) The statistical significance was evaluated by paired-samples *T* test. **(B)** Relative miR-191 expression in five human colorectal cancer cell lines (HCT116, RKO, HT29, SW480, DLD1 and Lovo) and human embryonic kidney 293T cells. U6 snRNA was used as an internal control. The data represents the means ± SDs.

Several reports indicated that miR-191 is up-regulated in human colorectal cancers by using high throughput sequencing [22–24]. Here, miR-191 expression was further analyzed in 16 paired colon and adjacent non-tumor colon tissues by way of real-time PCR. Our results demonstrated that miR-191 was up-regulated in the majority of examined tumor tissues, with 10 of 16 (62.5%) tumor tissues displaying a more than 38% increase, which suggested a probable ‘oncomiR’ role of miR-191 in colorectal cancer. Notably, there were three colon cancer tissues displayed a 3-fold down-regulation of miR-191 expression. (Figure 1A) We next examined miR-191 expression in five human colorectal cancer cell lines (HCT116, RKO, HT29, SW480, DLD1 and Lovo) and HEK293T cells by quantitative PCR. miR-191 was expressed in all six cell lines, and HCT116 displayed a higher expression level of miR-191 (Figure 1B). So, we used HCT116 cells as a model to investigate the effect of miR191 on cell growth and proliferation.

### miR-191 promotes the tumorigenic features of colorectal cancer cells

**Figure 2:**
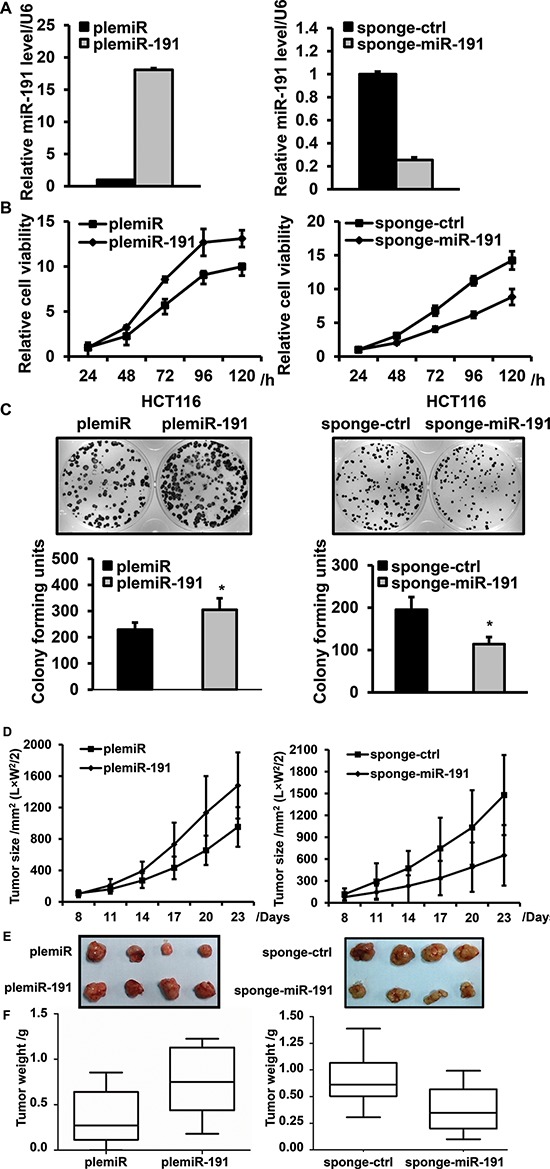
miR-191 promotes cell viability and proliferation **(A)** Confirmation of the level of miR-191 in stably transfected HCT116 cell lines by RT-PCR; cells transfected with the empty vector were used as a negative control. **(B)** The cell viability of HCT116 cells was determined by CCK8 assays after transfection of plemiR-191(left, B) and sponge-miR-191(right, B), at 24, 48, 72 96 and 120 hours. **(C)** Relative colony formation units of plemiR-191- (left, C) and sponge-miR-191-transfected (right, C) stable HCT116 cells. The lower panels indicate the quantification of the indicated relative colony-forming units (*n* = 3, *P < 0.05 versus plemiR-ctrl and sponge-ctrl). **(D)** The tumor volume of plemiR-191-/sponge-miR-191-treated mice 23 days after transplantation. **(E)** Representative images and **(F)** tumor weights of the isolated tumors from injected mice (*n* = 6 – 8). The data represents the means ± SDs.

To assess the role of miR-191 in the growth of CRC, stable miR-191-expressing cell lines were prepared using lenti-virus-mediated gene transfer, wherein the plemiR-191 and sponge-miR-191 were used as mediators for gain- and loss- of -function studies, respectively. The levels of miR-191 in the stable cell lines were determined by quantitative PCR, and our results demonstrated the effectiveness of this transfection (Figure 2A). Cell viability was measured using CCK8 assays, and we observed that plemiR-191-transfected HCT116 and RKO cells have a viability advantage over time, when compared with cells transfected with the plemiR-control. On the contrary, miR-191 inhibition by sponge-miR-191 decreased cell viability in HCT116, RKO, HT29 and SW480 cells (Figure 2B, Supplementary Figure 1A and Figure 2A). To examine the effect of miR-191 upon proliferation, the cells were seeded in 6-well plates (500 cells/well) for 14 days. The colony formation assay demonstrated a significant increase in the number of colony-forming units in the miR-191-transfected cells and a decrease in the number of clonies formed from sponge-miR-191-transfected cells (Figure 2C).

Next, a BALB/c nude mouse xenograft model was applied to evaluate the effect of miR-191 on tumorigenicity. The plemiR-191/plemiR-control or sponge-miR-191/sponge-control stable cell lines derived from HCT116 cells were subcutaneously injected into either the flank or forelimb armpits of nude mice. The tumor volume was measured regularly and tumor weights were recorded. Compared with the control group, miR-191-transfected cells revealed an advanced tumor formation and a significantly increase in tumor size and tumor weight. miR-191 inhibition effectively suppressed tumor growth in nude mice, as determined by the retarded tumor growth rate, reduced tumor volume and decreased tumor weight compared with the negative control (Figure 2D, 2E, 2F).

### miR-191 induces the G1-to-S cell-cycle transition

**Figure 3:**
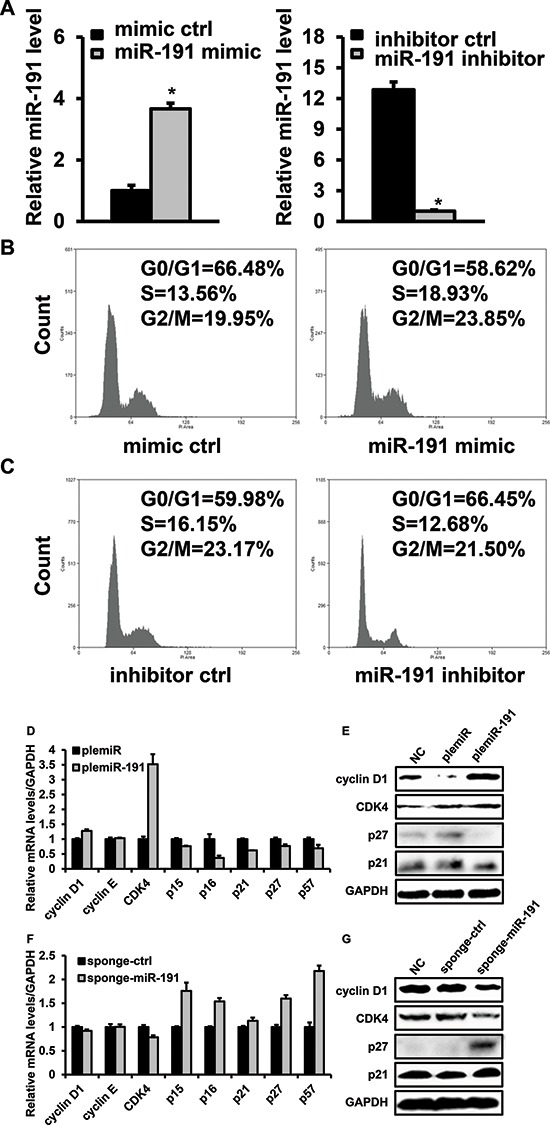
Effects of miR-191 on the cell cycle distribution of HCT116 cells **(A)** RT-PCR analysis confirmed the expression of miR-191 in miR-191 mimic-/inhibitior- transfected HCT116 cells; cells transfected with the corresponding control oligos were used as a negative control. **(B)** Cell cycle analysis of HCT116 cells transfected with the miR-191 mimic oligo. **(C)** Cell cycle analysis of HCT116 cells transfected with the miR-191 inhibitor oligo. **(D–G)** The mRNA levels of cyclin D1, cyclin E, CDK4, p15, p16, p21, p27 and p57 (D, F) and the protein levels of cyclin D1, CDK4, p21 and p27 (E, G) were assayed in plemiR-ctrl-/plemiR-191-transfected and sponge-ctrl-/sponge-miR-191-transfected HCT116 cells, respectively. GAPDH served as a loading control. The data represents the means ± SDs.

To elucidate the effect of miR-191 on cell cycle regulation, HCT116 cells transfected with miR-191 mimic or inhibitor were subjected to flow cytometry. First, we determined the miR-191 level to verify the effectiveness of the transfection. Quantitative PCR analysis showed that the expression of miR-191 increased by 3.6-fold and decreased by 12.8-fold following miR-191 mimic or inhibitor transfection, respectively. (Figure 3A) Cell cycle analysis showed a distinct decrease in the G1-phase cell population (66.48% vs. 58.62%) and an increase in the S-phase cell population (13.56% vs. 18.93%) in miR-191-mimic transfectants compared with the mimic control (Figure 3B). On the contrary, the miR-191 inhibitor restrained the G1-to-S cell cycle transition. (Figure 3C)

Cell cycle progression was controlled by a series of genes, including cell cycle progression regulators and cell cycle inhibitors such as p21, p15 and p16. Here, the mRNA and protein levels of cell cycle-related factors were detected by quantitative PCR and western blotting, respectively. We found that miR-191 induced the expression of CDK4, a key regulator in the G1-to-S cell cycle transition; however, the changes in the cyclin D1 and cyclin E levels were not significant. Notably, the levels of p15, p16, p21, p27 and p57, which are cyclin-dependent kinase inhibitors, were significantly decreased in the plemiR-191-transfected stable cell line (Figure 3D, 3E). In contrast, miR-191 inhibition by sponge-miR-191 led to a decrease in the level of CDK4 and an increase in the levels of p15, p16, p27 and p57 (Figure 3F, 3G).

### miR-191 promotes cell resistance to 5-Fu

**Figure 4:**
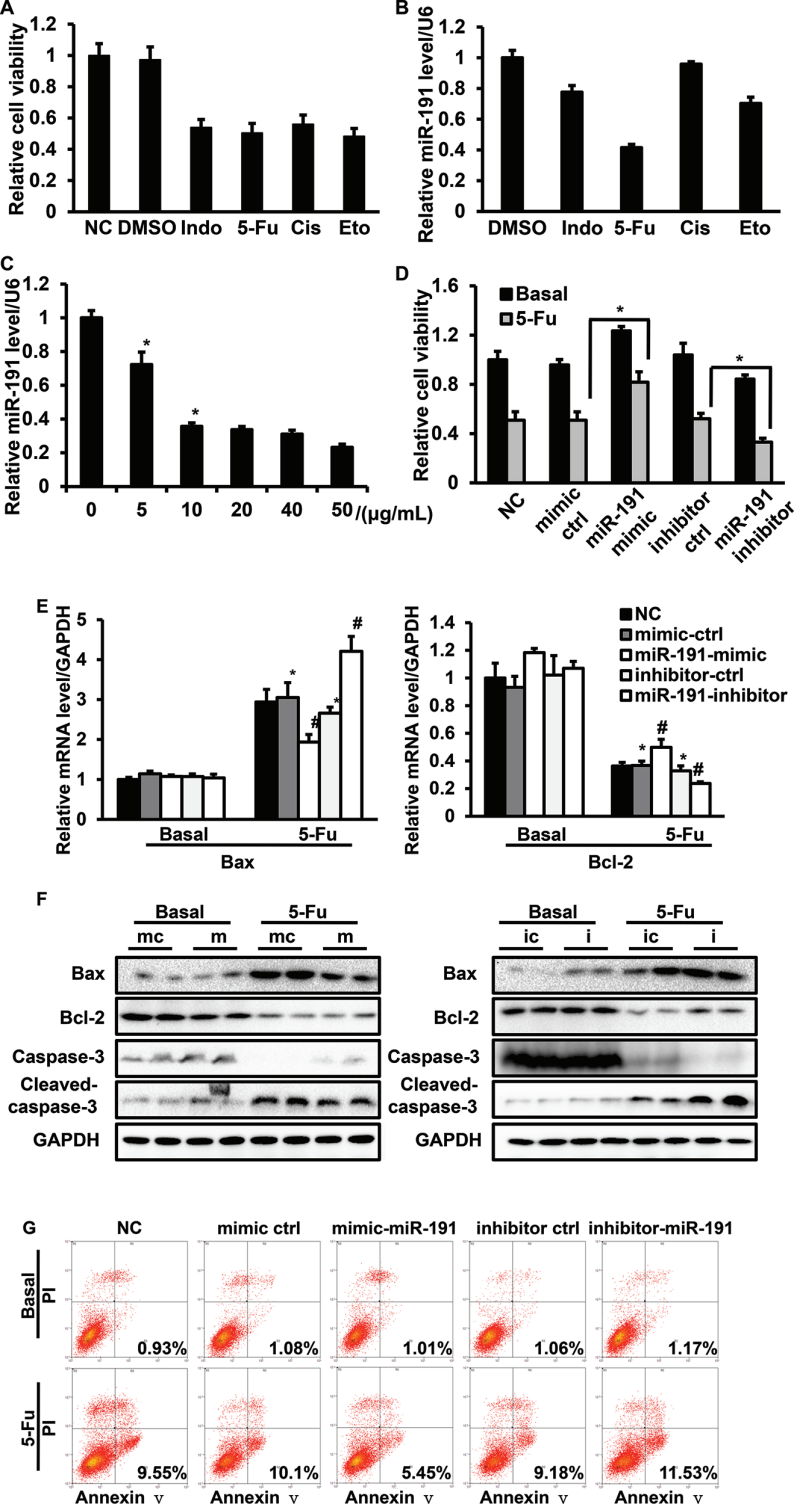
The involvement of miR-191 in the 5-Fu-induced cell apoptotic pathway in HCT116 cells HCT116 cells were treated with various commonly-used chemotherapeutic drugs, including 5-fluorouracil (5-Fu, 10 μg/ml), indomethacin (Indo, 10 μg/ml), cisplatin (Cis, 10 μg/ml) and etoposide (Eto, 2 μM). **(A)** Cell viability was assessed by CCK8 assays. **(B)** RT-PCR analysis of the relative expression of miR-191. **(C)** 5-Fu down-regulated miR-191 in a dose-dependent manner. HCT116 cells were treated with the indicated concentration of 5-Fu for 48 hours, total mRNA was isolated, and the miR-191 level was analyzed by RT-PCR (*P < 0.05 versus DMSO treated cells). HCT116 cells were transfected with the indicated oligos for 24 hours and then treated with 5-Fu (25 μg/ml) for another 24 hours. **(D)** Cell viability was assessed by CCK8 assays (*P < 0.05 versus 5-Fu treated mimic-ctrl/inhibitor-ctrl cells). **(E)** The mRNA levels of Bax and Bcl-2 was determined by RT-PCR (*P < 0.05 versus DMSO treated mimic-ctrl/inhibitor-ctrl cells, #*P* < 0.05 versus 5-Fu treated mimic-ctrl/inhibitor-ctrl cells). **(F)** The protein levels of Bax, Bcl-2, caspase-3 and cleaved-caspase-3 were detected by western blotting analysis in HCT116 cells transfected with mimic-ctrl/miR-191-mimic (left panel) and inhibitor-ctrl/miR-191-inhibitor (right panel) after DMSO or 5-Fu treatment. (mc, mimic ctrl; m, miR-191-mimic; ic, inhibitor ctrl; i, miR-191-inhibitor) **(G)** Cell apoptosis analysis of transfected cells after treatment. GAPDH served as a loading control. The data represents the means ± SDs.

5-Fu is a widely used anticancer drug, but due to its high-dose regimen in the clinic, the drug's side effects were observed. In our drug screening assay, we found that endogenous miR-191 was modulated by various drugs. Interestingly, although the changes of cell viability were similar when HCT116 cells were treated with different drugs, the alteration of the level of miR-191 was the most apparent in cells treated with 5-Fu (Figure 4A, 4B). We further demonstrated that 5-Fu decreased the endogenous miR-191 level in a dose-dependent manner (Figure 4C). The induction of the pro-apoptotic pathway by 5-Fu is crucial for its anticancer role, so we hypothesized that miR-191 might be involved in the 5-Fu induced cell apoptotic pathway. HCT116 cells were transfected with miR-191 mimic or inhibitor for 24 hours and then treated with 25 μg/ml 5-Fu for another 24 hours, and the cell viability was measured by CCK8. We observed an almost 50% decrease in cell viability when cells were treated with 5-Fu. Notably, when exposed to 5-Fu, cells transfected with the miR-191 mimic exhibited higher cell viability compared to cells transfected with the mimic control. The introduction of miR-191 inhibitor led to a significant decrease in cell viability when compared with the inhibitor control (Figure 4D).

To investigate the role of miR-191 in 5-Fu induced cell apoptosis, we performed quantitative PCR and western blotting to detect the mRNA and protein levels of pro-apoptotic Bax and anti-apoptotic Bcl-2, the markers of cell apoptosis. The mRNA level of Bax decreased in cells transfected with the miR-191-mimic and increased by 50% in cells transfected with the miR-191-inhibitor when compared with the control group. In contrast, the Bcl-2 level was higher in cells transfected with the miR-191-mimic and lower in cells transfected with the miR-191-inhibitor when compared with the corresponding control group (Figure 4E). The protein levels of Bax and Bcl-2 were consistent with the mRNA levels. Caspase-3 is an important downstream effecter of the cell apoptotic pathway and cleavage of caspase-3 was observed in HCT116 cells exposed to 5-Fu. Overexpression of miR-191 inhibited the cleavage of caspase-3, and suppression of miR-191 overtly induced the cleavage of caspase-3 (Figure 4F).

In addition, HCT116 cells transfected with the miR-191-mimic or miR-191-inhibitor were subjected to flow cytometry analysis. The apoptotic rate of cells transfected with miR-191-mimic was approximately 5.45%, which was significantly lower than of cells transfected with mimic-control oligo, which showed 10.1% apoptotic rates when exposed to 5-Fu. Compared with the inhibitor control, inhibition of miR-191 clearly increased the apoptotic rates of HCT116 cells treated with 5-Fu (Figure 4G). Taken together, miR-191 decreased the sensitivity of HCT116 cells to 5-Fu.

### miR-191 directly targets the 3′ UTR of C/EBPβ

**Figure 5:**
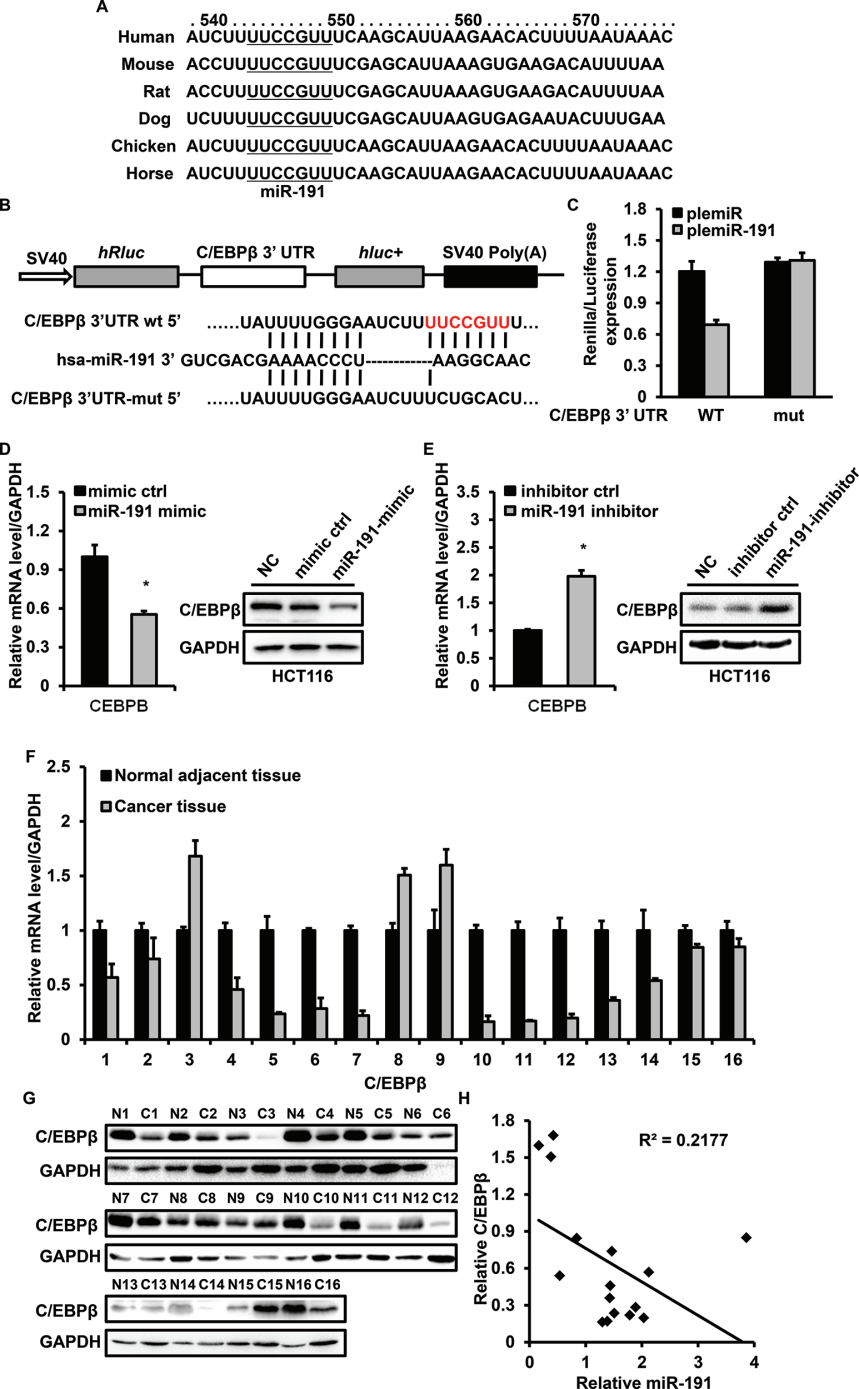
miR-191 down-regulates the expression of C/EBPβ **(A)** The predicted binding sites of miR-191 in the 3′ UTRs of C/EBPβ of different species. **(B)** Schematic description of wild type (WT) and mutated 3′ UTRs of the C/EBPβ mRNA. The WT and mutated 3′ UTR sequences (~400 bp) were cloned into the psiCHECK2 vector. **(C)** Luciferase analysis was used to detect the reporter activity. HCT116 cells in 24 well plates were co-transfected with 200 ng plemiR/plemiR-191 or psiCHECK2-WT 3′ UTR/psiCHECK2-mutated 3′ UTR (100 ng) for 48 hours and then subjected to luciferase assays according to the Materials and Methods. (D-E) miR-191 inhibits the mRNA and protein levels of C/EBPβ in HCT116 cells. HCT116 cells were transfected with the miR-191 mimic/mimic control **(D)** or miR-191 inhibitor/inhibitor control **(E)** for 48 hours and total mRNA and protein were extracted and analyzed by RT-PCR and western blotting analysis, respectively. GAPDH served as a loading control. **(F)** RT-PCR analysis of the relative expression of C/EBPβ in 16 colon cancer tissues. **(G)** Western blotting analysis of C/EBPβ in 16 colon cancer tissues. GAPDH served as a loading control. **(H)** Inverse correlation between miR-191 and C/EBPβ in colon cancer tissues. miR-191 level was normalized to the U6 level, and the C/EBPβ level was normalized to the GAPDH level. Statistical analysis was performed using Person's correlation coefficient analysis. The data represents the means ± SDs.

To explore the molecular mechanism responsible for the function of miR-191 in CRC, we used three publicly available databases (TargetScan, picTar and miRanda) to search for predicted direct target genes of miR-191. The predicted targets were arranged according to the binding probability score. The targets with high score and shared by the three databases were chosen. Seven candidate targets of miR-191 (unpublished and we are doing further research) were screened. C/EBPβ was chosen for further analysis, because previous reports have shown that C/EBPβ is an important regulator of cell growth [26, 27], cell apoptosis [28] and tumorigenicity [29]. miR-191 has conserved binding sites in the 3′UTRs of C/EBPβ of different species (Figure 5A). The wild-type (WT) or mutant 3′ UTR, in which the seed region was mutated to abolish miR-191 binding, was cloned into the psiCheck-2 plasmid (Figure 5B), and a dual-luciferase reporter system was employed to verify whether C/EBPBβ is a direct target of miR-191. The luciferase activity of the reporter containing the WT 3′ UTR of C/EBPβ was decreased in cells transfected with plemiR-191, whereas the activity of the mutant reporter was not significantly altered following plemiR-191 transfection (Figure 5C).

We further detected the expression of C/EBPβ in stable transfected HCT116 and RKO cells using quantitative PCR and western blotting. The results indicated that the mRNA and protein levels of C/EBPβ were impaired under conditions of miR-191 overexpression. Conversely, inhibition of miR-191 resulted in the up-regulation of the levels of C/EBPβ (Figure 5D, 5E and Supplementary Figure 1B and Figure 2B).

To determine the correlation between miR-191expression and the C/EBPβ level, we analyzed the mRNA and protein levels of C/EBPβ in the same set of specimens shown in Figure 1A, and the results showed that the miR-191 level was inversely correlated with C/EBPβ expression (Figure 5F–5H). These data suggested that C/EBPβ is a direct target of miR-191 in CRC.

### C/EBPβ inhibits cell proliferation and induces cell cycle arrest in CRC cell lines

**Figure 6:**
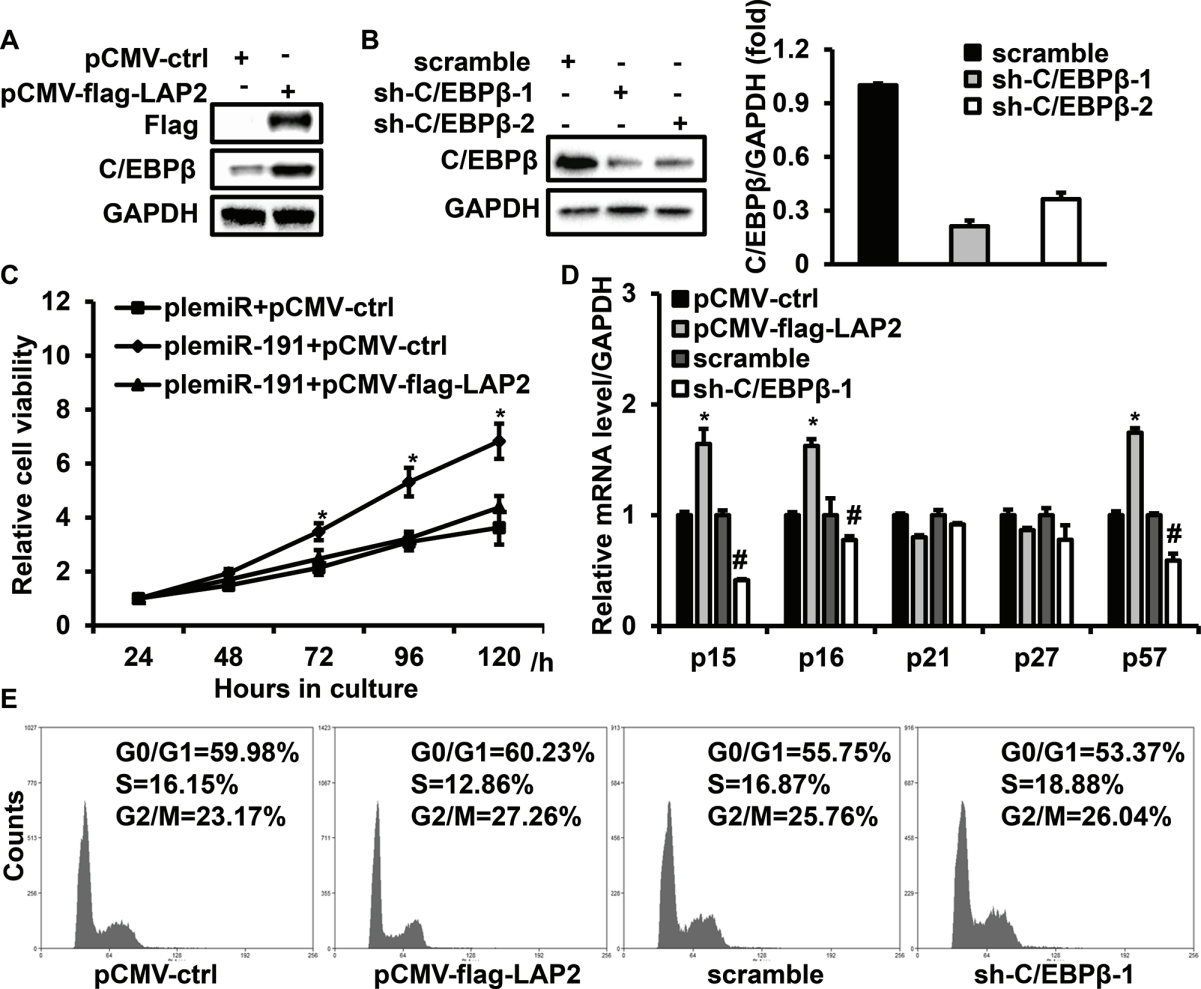
C/EBPβ is involved in miR-191 induced cell growth advantage HCT116 cells were transfected with flag-LAP2 or sh-C/EBPβ-1/2 for 48 hours and then analyzed by western blotting. Endogenous C/EBPβ expression was efficiently induced or repressed by transfection with flag-LAP2 **(A)** or sh-C/EBPβ-1/2. Right panel: Quantification of C/EBPβ levels **(B)**. GAPDH served as a loading control. **(C)** Ectopic expression of C/EBPβ abrogated the cell growth advantage caused by miR-191. HCT116 cells were transfected with the indicated expression vector for 48 hours and then analyzed by CCK8 assays (*P < 0.05 versus plemiR-191+pCMV-flag-LAP2 transfected cells). **(D)** The effects of C/EBPβ on the expression of p15, p16, p21, p27 and p57. Quantification of the mRNA levels of p15, p16, p21, p27 and p57 in HCT116 cells transfected with the indicated expression vectors for 48 hours. (*P < 0.05 versus pCMV-ctrl, #*P* < 0.05 versus scramble) **(E)** Cell cycle analysis of HCT116 cells transfected with the miR-191 mimic/mimic control or miR-191 inhibitor/inhibitor control for 48 hours. The data represents the means ± SDs.

Growth arrest induced by C/EBPβ is highly context specific [30–32]. To investigate the role of C/EBPβ in CRC, we performed loss- and gain-of-function studies for C/EBPβ. LAP2, the best studied and most transcriptionally active isoform of C/EBPβ, was given particular attention for its role of suppress cell cycle progression. [33, 34] HCT116 cells were transfected with pCMV-flag-LAP2 or sh-C/EBPβ-1/-2 and immunoblotting results showed the effectiveness of up-regulation and down-regulation of C/EBPβ by flag-LAP2 or sh-C/EBPβ-1 (Figure 6A, 6B). Ectopic expression of C/EBPβ diminished the role of miR-191 on cell viability (Figure 6C), and LAP2 transfection induced the expression of p16, p15 and p57. Although the alterations in the p21 and p27 levels were unconspicuous, the inhibition of C/EBPβ by shRNA led to decreased expression of p16, p15 and p57 (Figure 6D). In addition, the effect of C/EBPβ on the cell cycle transition was analyzed by flow cytometry. C/EBPβ overexpression led to G1 cell cycle arrest, while inhibition of endogenous C/EBPβ by sh-C/EBPβ-1 resulted in an increase in S phase (Figure 6E). These results further indicated that C/EBPβ is a direct target of miR-191.

## DISCUSSION

**Figure 7:**
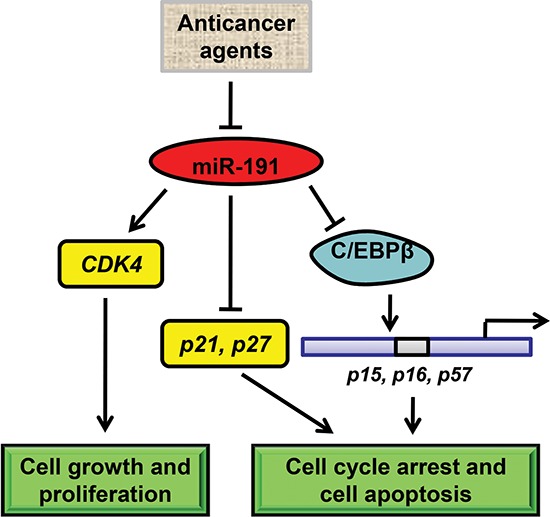
Schematic model of miR-191-mediated promotion of tumorigencity of colorectal cancer cells In colorectal cancer cells, various anticancer drugs regulate the expression of miR-191. miR-191 causes a number of genes to respond and adapt. On one hand, miR-191 induces the expression of CDK4 to increase cell growth. On the other hand, miR-191 suppresses the level of C/EBPβ, a tumor suppressor gene functions as a transcriptional activator of p15, p16 and p57, which are the key regulators of cell cycle and cell survival. As a result, miR-191 induces the cell cycle progression and tumor cell resistance to various stimuli.

In the present report, we showed that up-regulation of miR-191 was a frequent event in colon cancers and that this up-regulation increased cell viability and promoted cell proliferation and tumorigenicity of HCT116 cells. Anticancer reagents, such as 5-Fu and etoposide, inhibited the expression of miR-191 and sustained up-regulation of miR1-191 reduced cell susceptibility to 5-Fu. C/EBPβ was identified as a target of miR-191 and ectopic expression of C/EBPβ impaired the effect of miR-191 overexpression on CRC by activating expression of downstream genes, including p15, p16 and p57 (Figure 7). Our results suggest a crucial role of miR-191 in tumorigenesis of CRC and provide a therapeutic approach for CRC treatment.

Previous reports have shown that miR-191 is deregulated in various cancers and diseases and depending on the tissue and context conditions, miR-191 may exhibit specific functions [16–20]. Our study demonstrated a higher expression of miR-191 in colon cancer patients, which is consistent with previous reports [22]. Ectopic overexpression of miR-191 promoted cell proliferation in HCT116 cells and tumorigenicity *in vivo* in a nude mouse model. Furthermore, miR-191 mimic transfected cells displayed more DNA synthesis, while miR-191 inhibition delayed the G1-to-S cell cycle transition. Cyclin/cyclin-dependent kinases (Cyclin-CDK complexes) and CDK inhibitors (CKIs) coordinately control cell-cycle progression [35]. Our data showed that the level of CDK4, the key regulator of cell cycle progression, was altered when miR-191 was up- or down- regulated. Notably, the levels of p16, p15 and p57, which are important cell cycle inhibitors, were decreased when miR-191 was overexpressed. Di Leva et al. reported that cell cycle regulatory proteins such as CDK6 and CCND2 were established as targets of miR-191 in thyroid carcinoma and aggressive breast cancer and showed that miR-191-mediated down-regulation of CDK6 led to reduced cell proliferation [16, 20]. So, we determined the mRNA levels of CDK6 in HCT116 cells transfected with plemiR-191 or sponge-miR-191 and found that miR-191 also decreased the level of CDK6 in HCT116 cells (Supplementary Figure 3A). We considered that the tumor promoting role of miR-191 in CRC was primarily due to the suppression of cell cycle inhibitors. Further investigation is needed to establish the involvement of miR-191 in the alteration of the balance between cyclin/CDK complexes and CKIs.

It has been reported that miR-191 is a regulator of cell fate that inhibits cell apoptosis in colorectal carcinoma and hepatocellular carcinoma and miR-191 can be induced by various stimuli such as hypoxia and serum starvation [17, 20, 25]. 5-Fu is a widely used antimetabolite drug used for the treatment of digestive tumors, particularly for colorectal cancer. Our study revealed that endogenous miR-191 was regulated by 5-Fu in a dose-dependent manner, which is consistent with results of a previous study [36]. Our further study showed that miR-191 was involved in the 5-Fu-induced cell apoptotic pathway and that miR-191 overexpression increased cell resistance to 5-Fu-induced cell apoptosis. These results were partially consistent with the previous reports which showed that miR-191 inhibition induced cell apoptosis by upregulation of pro-apoptotic TIMP3. Interestingly, we found that the level of miR-191 was down-regulated by etoposide, a commonly used anticancer agent and an inducer of DNA double-strand breaks and cell apoptosis. Xi et al. reported that miR-191 was up-regulated in tumors with a p53 deletion [22]; therefore, it is possible that inhibition of miR-191 renders the cells more susceptible to p53-dependent stress responses. Considering the high mutation rate of p53 in human CRC, it would be intresteing to see whether introduction of miR-191 into CRC cells can modulate p53 activity and uncover the mechanism of miR-191 action. Here, we conclude that miR-191 is an anti-apoptotic gene in a colorectal carcinoma, in 5-Fu-induced cell apoptotic model.

CCAAT/enhancer-binding protein β (C/EBPβ) is a member of the C/EBP family of transcription factors [37, 38]. C/EBPβ has been shown to regulate cell proliferation, differentiation and cell apoptosis in a variety of cellular systems [39–41]. In breast cancer, C/EBPβ was identified as a direct target of miR-155, which is an oncogenic microRNA involved in cell proliferation and the inflammation response [42]. In this study, we demonstrated that miR-191 could directly bind to the 3′UTR region of C/EBPβ and decrease its mRNA and protein levels, which suggests a novel signaling mechanism wherein miR-191 functions as an ‘onco-miR’. Forced expression of exogenous C/EBPβ can induce cell apoptosis in various malignant cells and growth arrest induced by C/EBPβ is highly context-specific [40, 43, 44]. Overexpression of C/EBPβ arrests cells at or near the G1-S boundary in HepG2 and epidermal keratinocytes [30, 45]. While, in several cases, C/EBPβ displays growth-promoting activity [31, 32]. p21 and p57 were reported as regulated targets of C/EBPβ in MCF7 and chondrocytes [43, 46]. Our findings provide evidence that C/EBPβ expression was low in colon tumor patients and that it functioned as a tumor suppressor in HCT116 cells by suppressing of cell viability and inducing of cell apoptosis, which is consistent with results of a previous study showing that administration of full-length wild-type C/EBPβ significantly suppressed the growth of colon tumors by inducing tumor-cell apoptosis in a nude mouse model [29]. Furthermore, the expression of miR-191 was negatively related to the level of C/EBPβ in 16-paired tumor and non-tumor patient samples. Collectively, this evidence strongly suggests that C/EBPβ is regulated by miR-191 in human colorectal cancer, although the signal transduction is still not well understood.

The present study provides evidence indicating that inhibition of miR-191 suppresses the proliferation of colorectal cells and tumorigenicity *in vivo*, and that miR-191-down-regulated cells are more sensitive to 5-Fu-induced cell apoptosis. Due to the regulation of C/EBPβ and downstream signaling by miR-191, the specific inhibition of miR-191 or when in combination with 5-Fu may be useful for the treatment of human colorectal cancer. However, considering technology and the number of patients limitations in our study, cancer or disease specific mouse models, such as tissue-specific transgenic or knockout mice, would be extremely helpful to verify miR-191 therapeutic potential.

## MATERIALS AND METHODS

### Tissue specimens

CRC and adjacent non-tumor colon tissues were collected from patients undergoing resection of CRC. Tissue samples were immediately frozen in liquid nitrogen and stored until total RNAs or proteins were extracted. Informed consent was obtained at the Tongji Medical College Huazhong University of Science & Technology in Wuhan, China. None of the patients received chemotherapy prior to colectomy. All patients were unrelated ethnic Han Chinese who lived in Southeast China; the tissues were characterized using immunochemistry methods. The relevant characteristics of the studied subjects are shown in Supplementary Table 1.

### Cell culture and reagents

The human embryonic kidney 293T cell line (HEK293T) and six human colorectal cancer cell lines (HCT116, RKO, HT29, SW480, DLD1 and Lovo) were purchased from American Type Culture Collection (ATCC, Manassas, VA). The cell lines HCT116, RKO, HT29, SW480, DLD1 and Lovo were grown in McCoy's 5A medium (AppliChem, A1324, 9050, Darmstadt, Germany) supplemented with 10% fetal bovine serum (FBS, Gibco, Carlsbad, CA, USA) and 1% penicillin–streptomycin (HyClone, Logan, Utah, USA) at 37°C, in 5% CO_2_. The HEK293T cell line was maintained in Dulbecco's modified Eagle's medium (DMEM, HyClone, Logan, Utah, USA) supplemented with 10% fetal bovine serum and 1% penicillin–streptomycin at 37°C in 5% CO_2_. 5-Fluorouracil (5-Fu), indomethacin (Indo), cisplatin (Cis) and etoposide (Eto) were obtained from Sigma Chemical Co. (St. Louis, MO, USA)

### Plasmids

Has-miR-191-5p mimic, has-miR-191-5p inhibitor and the corresponding mimic/inhibitor control oligo were purchased from Guangzhou RiboBio Co., LTD. (Guangzhou, China) The human miRNA expression vectors plemiR and plemiR-191 were kindly provided by Dr. Yendamuri, Sai (Department of Thoracic Surgery, Roswell Park Cancer Institute, Buffalo, USA) [47]. An miR-191 sponge(sponge-miR-191) was constructed using the methods described by Margaret S Ebert [48]. The sponge sequence, encoding ten repeats of reverse complementary sequence of mature miR-191 was synthesized by Sunny Biotechnology Co. (Shanghai, China) and then the sequence was ligated to the pSicoR vector after digestion by BamH1 and Xho1. The human C/EBPβ expression vector pCMV-flag-LAP2 was purchased from Addgene (ID 15738). The shRNA sequences against C/EBPβ were as follows: sequence 1, CCCGTGGTGTTATTTAAAGAA; sequence 2, CCTGCCTTTAAATCCATGGAA, and the scramble shRNA sequence was AATTCTCCGAACGTGTCACGT. shRNAs were ligated into the pLKO.1 puro vector (Addgene, # 8453). All plasmids were sequenced to verify the inserted targets. HCT116 cells were transfected with the oligos (100 nmol/L) or indicated plasmids, using Lipofectamine2000^™^ (Invitrogen, Carlsbad, CA, USA) in OptiMEM medium according to the manufacturer's instructions. The OptiMEM media (Invitrogen, Carlsbad, CA, USA) was replaced with cell growth media after 8 hours of transfection.

### Cell viability and colony formation assay

Cell Counting Kit-8 (CCK8) assays were performed to analyze cellular proliferation and activity. HCT116 cells transfected with plemiR-191 or pSicoR-sponge-miR-191 (100 μL) were seeded at a density of 2 × 10^3^ cells/well in 96-well plates. At 24, 48, 72, 96 and 120 hours, 10 μL of CCK8 solution (Dojindo Laboratories, Kumamoto, Japan) was added to each well of the plate and incubated for 1 hour. Cell viability was then determined using a spectrophotometer set (ELx800, BioTek, USA) at a wavelength of 450 nm. For colony formation assays, plemiR-191- or pSicoR-sponge-miR-191-transfected HCT116 cells were cultured in a 6-well plate at 500 cells per well and grown for 10-14 days. After fixation by 4% paraformaldehyde (Electron Microscopy Sciences 16% Paraformaldehyde Cat.15700, diluted into PBS) for 30 minutes, the colonies were stained with 0.1% Crystal Violet for 15 minutes and washed. The colonies were then photographed and counted using the Image J software.

### Cell cycle and cell apoptosis analysis

HCT116 cells were transfected with the indicated oligos or plasmids in 6-well plates. After 48 hours of transfection, the cells were collected and washed with PBS. For cell cycle analysis, the cells were fixed with 70% ethanol overnight at −20°C, washed with PBS, resuspended with 400 μl PBS and then incubated with 100 μg/ml RNaseA (Sigma) for 30 minutes at 37°C and with 50 μg/ml propidium iodide (PI) (Sigma, St Louis, MO) for another 10 minutes. After incubation, the cells were subjected to DNA content analysis using a FACSCalibur (Beckman Coulter, Fullerton, CA) and the results were analyzed with the Summit v4.3 software. Apoptosis was evaluated by Annexin V-FITC (BD Biosciences, 556419) and PI (BD Biosciences, 556463) staining according to the manufacturer's protocol, followed by flow cytometry analysis. In brief, HCT116 cells (1 × 10^5^) were collected, washed with ice-cold PBS and resuspended in 100 μl binding buffer. Then, 2 μl of Annexin V-FITC and 5 μl of PI were added to the cells, the cells were incubated for 15 minutes at RT in the dark, and an additional 400 μl of binding buffer was added to the reaction prior to analysis. The results were analyzed with the Summit v4.3 software.

### Tumorigenicity assays in nude mice

All experimental procedures involving animals were performed in accordance with the Guide for the Care and Use of Laboratory Animals (NIH publications Nos. 80–23, revised 1996) and were approved by the Animal Care and Use Committee of Wuhan University. Female athymic nude mice were bought from (HFKBio, Peking, China). PlemiR-191 or pSicoR-sponges-miR-191 transfected HCT116 cells and the corresponding control cells (5 × 106) were suspended in 200 μl PBS and then injected subcutaneously into either the flank or forelimb armpits of the same female, 5−6-week-old BALB/c athymic nude mouse (HFKBio, Peking, China). Seven to ten female mice were included in the experiments. Tumor growth was examined every three days, and the tumors were removed and weighed 23 days after injection. The tumor volume (V) was monitored using measuring the length (L) and width (W) of the tumor with calipers and was calculated using the formula V = (L × W^2^) × 0.5.

### Lentiviral transduction

According to the Addgene protocol (http://www.addgene.org/tools/protocols/plko/), human 293T cells cultured in a 6 cm dish were cotransfected with the lentiviral packaging vectors 1 μg of psPAX2 (Addgene, #12260), 2 μg of pMD2.G (Addgene, #12259), and 3 μg of the indicated plasmids to produce lentiviral particles. Sixty hours after transfection, the media were collected and filtered with a 0.45 μm filter (Nalgene, Rochester, NY, USA). Viral supernatant mixed with 5 μg/mL polybrene (Sigma Chemical Co., St. Louis, Mo.) was used to infect HCT116 cells for 12 hours, and was then replaced with fresh media.

### Reporter assays

For the C/EBPβ 3′UTR reporter assays, experiments were performed on the HCT116 cell line. The 3′UTR fragment of C/EBPβ was amplified from human genomic DNA and subcloned into the XhoI and NotI sites of the psiCHECK2 vector (Promega, Madison, WI). The sequences of the primers used for PCR amplification were as follows: forward primer: 5′-CCCTCGAG GCAACCCACGTGTAACTGTC-3′ and reverse primer 5′-ATAAGAATGCGGCCGCCACCC AACCACCAAAACCTC-3′. Mutagenesis of the miR-191 seed sequence was performed using the following primers: forward primer: 5′-AAGGGAATCTTTCT GCACTCAAGCAT-3′ and reverse primer 5′-ATGC TTGAGTGCAGAAAGATTCCCTT-3′. HCT116 cells were transfected with the reporter plasmid (100 ng) and plemiR-191/plemiR-ctrl (200 ng), and reporter assays were performed 24 hours after transfection using the Dual Luciferase kit (Promega, Madison, WI).

### RNA quantification

Total RNA was extracted using TRIzol reagent (Takara Biotechnology, Dalian, China) according to the manufacturer's protocol, and the concentrations of the RNA were determined with a NanoDrop instrument (NanoDrop Technologies). Complementary DNA was prepared with the use of a First Strand cDNA Synthesis Kit (Roche Diagnostics, Mannheim, Germany) and stem-loop RT for mature miRNAs was performed according to the manufacturer's protocol. (Roche) All reagents for stem-loop RT were obtained from Roche (Roche Diagnostics, Mannheim, Germany) and RiboBio (Guangzhou, China). The U6 snRNA was used as an internal control. Real-time PCR was performed using the FastStart Universal SYBR Green Master protocol (ROCHE, 04913850001) with the ABI PRISM 7500 system (Applied Biosystems, Forster City, Calif). For microRNA PCR, the reactions were incubated in a 96-well plate at 95°C for 10 minutes, followed by 40 cycles of 95°C for 15 seconds and 60°C for 1 minute. All reactions were run in triplicate, and the relative gene expression was calculated using the comparative threshold cycle (Ct) method (relative gene expression = 2^−(ΔCtsample-ΔCtcontrol)^). The following primers were used for qPCR: C/EBPβ sense, 5′-TTCAAGCAGCTGCCCGAGCC-3′; C/EBPβ antisense, 5′-GCCAAGTGCCCCAGTGCCAA-3′ (Refer to Gibellini et al [49]); cyclin D1 sense, 5′-CCGTCCATGCGGAAGATC-3′; cyclin D1 antisense, 5′-CCTCCTCCTCGCACTTCTGT-3′; cyclin E1 sense, 5′-TTTCTTGAGCAACACCCT-3′; cyclin E1 antisense, 5′-GTCACATACGCAAACTGG-3′; CDK4 sense, 5′-GAAACTCTGAAGCCGACCAG-3′; CDK4 antisense, 5′-AGGCAGAGATTCGCTTGTGT-3′; CDK6 sense, 5′-CCGTGGATCTCTGGAGTGTT-3′; CDK6 antisense, 5′-CTCAATTGGTTGGGCAGATT-3′; p15 sense, 5′-ATGCGCGAGGAGAACAAG-3′; p15 antisense, 5′-CTCCCGAAACGGTTGACTC-3′; p16 sense, 5′-CTTCCTGGACACGCTGGT-3′; p16 antisense, 5′-ATCTATGCGGGCATGGTTAC-3′; p21 sense, 5′-GAGCGATGGAACTTCGACTT-3′; p21 antisense, 5′-CAGGTCCACATGGTCTTCCT-3′; p27 sense, 5′-GGTCTGCAAGTGGATGATGA-3′; p27 antisense, 5′-ATAATGTTGCAGCCCAGCAG-3′; p57 sense, 5′-CACGATGGAGCGTCTTGTC-3′; p57 antisense, 5′-CCTGCTGGAAGTCGTAATCC-3′; Bax sense, 5′-GAGGATGATTGCCGCCGTGGACA-3′; Bax antisense, 5′-GGTGGGGGAGGAGGCTTGAGG-3′; Bcl-2 sense, 5′-ATGTGTGTGGAGAGCGTCAACC-3′; Bcl-2 antisense, 5′-TGAGCAGAGTCTTCAGAGACAGCC-3′; GAPDH sense, 5′-GAAGGTGAAGGTCGGAGTC-3′; and GAPDH antisense, 5′-GAAGATGGTGATGGGATT TC-3′.

### Western blotting analysis

The cells were lysed in RIPA buffer (1% v/v NP40, 0.5% w/v sodiumdeoxycholate, 0.1% w/v SDS) containing complete protease inhibitors (Roche Applied Sciences) in an ice bath for 30 minutes, and then centrifuged at 13,000 × g for 30 minutes at 4°C. The protein concentration was then determined using the Pierce^®^ BCA Protein Assay Kit (Pierce, 23225). Equal amounts of protein (30-150 ug) were separated by 12% SDS-PAGE and transferred to PVDF membranes (PVDF, Millipore, cat# IPVH00010, Merck KgaA, Darmstadt, Germany). The membranes were blocked with 5% non-fat dry milk in TBST (20 mM Tris-HCl, pH 7.5, 150 mM NaCl, 0.1% Tween-20) for 1 hour at room temperature and then incubated with primary antibody on a rocking platform overnight at 4°C, followed by incubation with a horseradish peroxidase-conjugated secondary antibody. Protein bands were visualized by incubating the membranes with the SuperSignal chemiluminescence kit (Merck Millipore). Finally, Bio-Rad's ChemiDoc XRS+ imaging system was used for signal detection. Protein expression levels were normalized to GAPDH as a loading control. The antibodies used in this study were as follows: Rabbit monoclonal antibodies against Bax (#5023), Bcl-2 (#4223), caspase 3 (#9665), cleaved-caspase-3(#9661), p21 (#2947) and GAPDH (#5174) and; rabbit polyclonal antibody against C/EBPβ (#3082) were obtained from Cell Signaling Technology (Beverly, MA). Rabbit anti-p27 (sc-528), anti-CDK4 (sc-260), and anti-cyclin D (sc-753) were from Santa Cruz Biotechnology. Peroxidase-conjugated Goat Anti-Mouse IgG (H+L (115-035-003 and Peroxidase-conjugated Goat Anti-Rabbit IgG (H+L) (111-035-003) were purchased from Jackson ImmunoResearch. (Jackson, West Baltimore Pike West Grove, PA, USA)

### Statistical analysis

The SPSS 19 software was used for statistical analysis, and the data are presented as the means ± SDs. Differences between groups were assessed by one-way ANOVA, and statistical significance was determined by Student's *t*-test in some experiments. Differences with *P* values of less than 0.05 were considered significant.

## SUPPLEMENTARY FIGURES AND TABLE



## References

[1] Bartel DP (2004). MicroRNAs: genomics, biogenesis, mechanism, and function. Cell.

[2] Baek D, Villen J, Shin C, Camargo FD, Gygi SP, Bartel DP (2008). The impact of microRNAs on protein output. Nature.

[3] Carthew RW (2006). Gene regulation by microRNAs. Curr Opin Genet Dev.

[4] Calin GA, Croce CM (2006). MicroRNA signatures in human cancers. Nat Rev Cancer.

[5] Lu J, Getz G, Miska EA, Alvarez-Saavedra E, Lamb J, Peck D, Sweet-Cordero A, Ebert BL, Mak RH, Ferrando AA, Downing JR, Jacks T, Horvitz HR (2005). MicroRNA expression profiles classify human cancers. Nature.

[6] Lee YS, Dutta A (2007). The tumor suppressor microRNA let-7 represses the HMGA2 oncogene. Genes Dev.

[7] Bonci D, Coppola V, Musumeci M, Addario A, Giuffrida R, Memeo L, D'Urso L, Pagliuca A, Biffoni M, Labbaye C, Bartucci M, Muto G, Peschle C (2008). The miR-15a-miR-16-1 cluster controls prostate cancer by targeting multiple oncogenic activities. Nat Med.

[8] He L, Thomson JM, Hemann MT, Hernando-Monge E, Mu D, Goodson S, Powers S, Cordon-Cardo C, Lowe SW, Hannon GJ, Hammond SM (2005). A microRNA polycistron as a potential human oncogene. Nature.

[9] He L, He X, Lim LP, de Stanchina E, Xuan Z, Liang Y, Xue W, Zender L, Magnus J, Ridzon D, Jackson AL, Linsley PS, Chen C (2007). A microRNA component of the p53 tumour suppressor network. Nature.

[10] Siegel R, Naishadham D, Jemal A (2013). Cancer statistics. Ca-Cancer J Clin.

[11] Zhang J, Fei B, Wang Q, Song M, Yin Y, Zhang B, Ni S, Guo W, Bian Z, Quan C, Liu Z, Wang Y, Yu J (2014). MicroRNA-638 inhibits cell proliferation, invasion and regulates cell cycle by targeting tetraspanin 1 in human colorectal carcinoma. Oncotarget.

[12] Fang L, Li H, Wang L, Hu J, Jin T, Wang J, Yang BB (2014). MicroRNA-17-5p promotes chemotherapeutic drug resistance and tumour metastasis of colorectal cancer by repressing PTEN expression. Oncotarget.

[13] Hara T, Jones MF, Subramanian M, Li XL, Ou O, Zhu Y, Yang Y, Wakefield LM, Hussain SP, Gaedcke J, Ried T, Luo J, Caplen NJ (2014). Selective targeting of KRAS-mutant cells by miR-126 through repression of multiple genes essential for the survival of KRAS-mutant cells. Oncotarget.

[14] Perilli L, Vicentini C, Agostini M, Pizzini S, Pizzi M, D'Angelo E, Bortoluzzi S, Mandruzzato S, Mammano E, Rugge M, Nitti D, Scarpa A, Fassan M (2014). Circulating miR-182 is a biomarker of colorectal adenocarcinoma progression. Oncotarget.

[15] Hrasovec S, Glavac D (2012). MicroRNAs as Novel Biomarkers in Colorectal Cancer. Front Genet.

[16] Hui AB, Shi W, Boutros PC, Miller N, Pintilie M, Fyles T, McCready D, Wong D, Gerster K, Waldron L, Jurisica I, Penn LZ, Liu FF (2009). Robust global micro-RNA profiling with formalin-fixed paraffin-embedded breast cancer tissues. Lab Invest.

[17] Elyakim E, Sitbon E, Faerman A, Tabak S, Montia E, Belanis L, Dov A, Marcusson EG, Bennett CF, Chajut A, Cohen D, Yerushalmi N (2010). hsa-miR-191 Is a Candidate Oncogene Target for Hepatocellular Carcinoma Therapy. Cancer Res.

[18] Colamaio M, Borbone E, Russo L, Bianco M, Federico A, Califano D, Chiappetta G, Pallante P, Troncone G, Battista S, Fusco A (2011). miR-191 Down-Regulation Plays a Role in Thyroid Follicular Tumors through CDK6 Targeting. J Clin Endocr Metab.

[19] Garzon R, Volinia S, Liu CG, Fernandez-Cymering C, Palumbo T, Pichiorri F, Fabbri M, Coombes K, Alder H, Nakamura T, Flomenberg N, Marcucci G, Calin GA (2008). MicroRNA signatures associated with cytogenetics and prognosis in acute myeloid leukemia. Blood.

[20] Nagpal N, Ahmad HM, Molparia B, Kulshreshtha R (2013). MicroRNA-191, an estrogen-responsive microRNA, functions as an oncogenic regulator in human breast cancer. Carcinogenesis.

[21] Di Leva G, Piovan C, Gasparini P, Ngankeu A, Taccioli C, Briskin D, Cheung DG, Bolon B, Anderlucci L, Alder H, Nuovo G, Li M, Iorio MV (2013). Estrogen mediated-activation of miR-191/425 cluster modulates tumorigenicity of breast cancer cells depending on estrogen receptor status. PLoS Genet.

[22] Xi Y, Formentini A, Chien M, Weir DB, Russo JJ, Ju J, Kornmann M, Ju J (2006). Prognostic Values of microRNAs in Colorectal Cancer. Biomarker insights.

[23] Volinia S, Calin GA, Liu CG, Ambs S, Cimmino A, Petrocca F, Visone R, Iorio M, Roldo C, Ferracin M, Prueitt RL, Yanaihara N, Lanza G (2006). A microRNA expression signature of human solid tumors defines cancer gene targets. Proc Natl Acad Sci U S A.

[24] Cummins JM, He Y, Leary RJ, Pagliarini R, Diaz LA, Sjoblom T, Barad O, Bentwich Z, Szafranska AE, Labourier E, Raymond CK, Roberts BS, Juhl H (2006). The colorectal microRNAome. Proc Natl Acad Sci U S A.

[25] Qin S, Zhu Y, Ai F, Li Y, Bai B, Yao W, Dong L (2014). MicroRNA-191 correlates with poor prognosis of colorectal carcinoma and plays multiple roles by targeting tissue inhibitor of metalloprotease 3. Neoplasma.

[26] Luedde T, Duderstadt M, Streetz KL, Tacke F, Kubicka S, Manns MP, Trautwein C (2004). C/EBP beta isoforms LIP and LAP modulate progression of the cell cycle in the regenerating mouse liver. Hepatology.

[27] Pal R, Janz M, Galson DL, Gries M, Li S, Johrens K, Anagnostopoulos I, Dorken B, Mapara MY, Borghesi L, Kardava L, Roodman GD, Milcarek C (2009). C/EBPbeta regulates transcription factors critical for proliferation and survival of multiple myeloma cells. Blood.

[28] Shimizu Y, Kishimoto T, Ohtsuka M, Kimura F, Shimizu H, Yoshidome H, Miyazaki M (2007). CCAAT/enhancer binding protein-beta promotes the survival of intravascular rat pancreatic tumor cells via antiapoptotic effects. Cancer Sci.

[29] Sun L, Fu BB, Liu DG (2005). Systemic delivery of full-length C/EBP beta/liposome complex suppresses growth of human colon cancer in nude mice. Cell Res.

[30] Buck M, Turler H, Chojkier M (1994). LAP (NF-IL-6), a tissue-specific transcriptional activator, is an inhibitor of hepatoma cell proliferation. EMBO J.

[31] Bundy LM, Sealy L (2003). CCAAT/enhancer binding protein beta (C/EBPbeta)-2 transforms normal mammary epithelial cells and induces epithelial to mesenchymal transition in culture. Oncogene.

[32] Wessells J, Yakar S, Johnson PF (2004). Critical prosurvival roles for C/EBP beta and insulin-like growth factor I in macrophage tumor cells. Mol Cell Biol.

[33] Calkhoven CF, Muller C, Leutz A (2000). Translational control of C/EBPalpha and C/EBPbeta isoform expression. Genes Dev.

[34] Nerlov C (2010). Transcriptional and translational control of C/EBPs: the case for "deep" genetics to understand physiological function. Bioessays.

[35] Besson A, Dowdy SF, Roberts JM (2008). CDK inhibitors: cell cycle regulators and beyond. Dev Cell.

[36] Zhou J, Zhou Y, Yin B, Hao W, Zhao L, Ju W, Bai C (2010). 5-Fluorouracil and oxaliplatin modify the expression profiles of microRNAs in human colon cancer cells *in vitro*. Oncol Rep.

[37] Akira S, Isshiki H, Sugita T, Tanabe O, Kinoshita S, Nishio Y, Nakajima T, Hirano T, Kishimoto T (1990). A nuclear factor for IL-6 expression (NF-IL6) is a member of a C/EBP family. EMBO J.

[38] Ramji DP, Foka P (2002). CCAAT/enhancer-binding proteins: structure, function and regulation. Biochem J.

[39] Grimm SL, Rosen JM (2003). The role of C/EBPbeta in mammary gland development and breast cancer. J Mammary Gland Biol.

[40] Lekstrom-Himes J, Xanthopoulos KG (1998). Biological role of the CCAAT/enhancer-binding protein family of transcription factors. J Biol Chem.

[41] Umek RM, Friedman AD, McKnight SL (1991). CCAAT-enhancer binding protein: a component of a differentiation switch. Science.

[42] Johansson J, Berg T, Kurzejamska E, Pang MF, Tabor V, Jansson M, Roswall P, Pietras K, Sund M, Religa P, Fuxe J (2013). MiR-155-mediated loss of C/EBPbeta shifts the TGF-beta response from growth inhibition to epithelial-mesenchymal transition, invasion and metastasis in breast cancer. Oncogene.

[43] Pal P, Lochab S, Kanaujiya J, Sanyal S, Trivedi AK (2010). Ectopic expression of hC/EBPs in breast tumor cells induces apoptosis. Mol Cell Biochem.

[44] Zhu MS, Liu DG, Cheng HQ, Xu XY, Li ZP (1997). Expression of exogenous NF-IL6 induces apoptosis in Sp2/0-Ag14 myeloma cells. DNA Cell Biol.

[45] Zhu S, Yoon K, Sterneck E, Johnson PF, Smart RC (2002). CCAAT/enhancer binding protein-beta is a mediator of keratinocyte survival and skin tumorigenesis involving oncogenic Ras signaling. Proc Natl Acad Sci U S A.

[46] Hirata M, Kugimiya F, Fukai A, Ohba S, Kawamura N, Ogasawara T, Kawasaki Y, Saito T, Yano F, Ikeda T, Nakamura K, Chung UI, Kawaguchi H (2009). C/EBPbeta Promotes transition from proliferation to hypertrophic differentiation of chondrocytes through transactivation of p57. PloS one.

[47] Patnaik SK, Kannisto E, Yendamuri S (2010). Overexpression of microRNA miR-30a or miR-191 in A549 lung cancer or BEAS-2B normal lung cell lines does not alter phenotype. PloS one.

[48] Ebert MS, Neilson JR, Sharp PA (2007). MicroRNA sponges: competitive inhibitors of small RNAs in mammalian cells. Nat Methods.

[49] Gibellini D, Alviano F, Miserocchi A, Tazzari PL, Ricci F, Clo A, Morini S, Borderi M, Viale P, Pasquinelli G, Pagliaro P, Bagnara GP, Re MC (2011). HIV-1 and recombinant gp120 affect the survival and differentiation of human vessel wall-derived mesenchymal stem cells. Retrovirology.

