# Supplementary material for: miR-191 promotes tumorigenesis of human colorectal cancer through targeting C/EBPβ
Source: Oncotarget. 2014 Dec 27;6(6):4144–58. doi: 10.18632/oncotarget.2864 (PMC4414178; doi:10.18632/oncotarget.2864)
Supplement: Supplementary file 1 [file oncotarget-06-4144-s001.pdf]

## SUPPLEMENTARY FIGURES AND TABLE

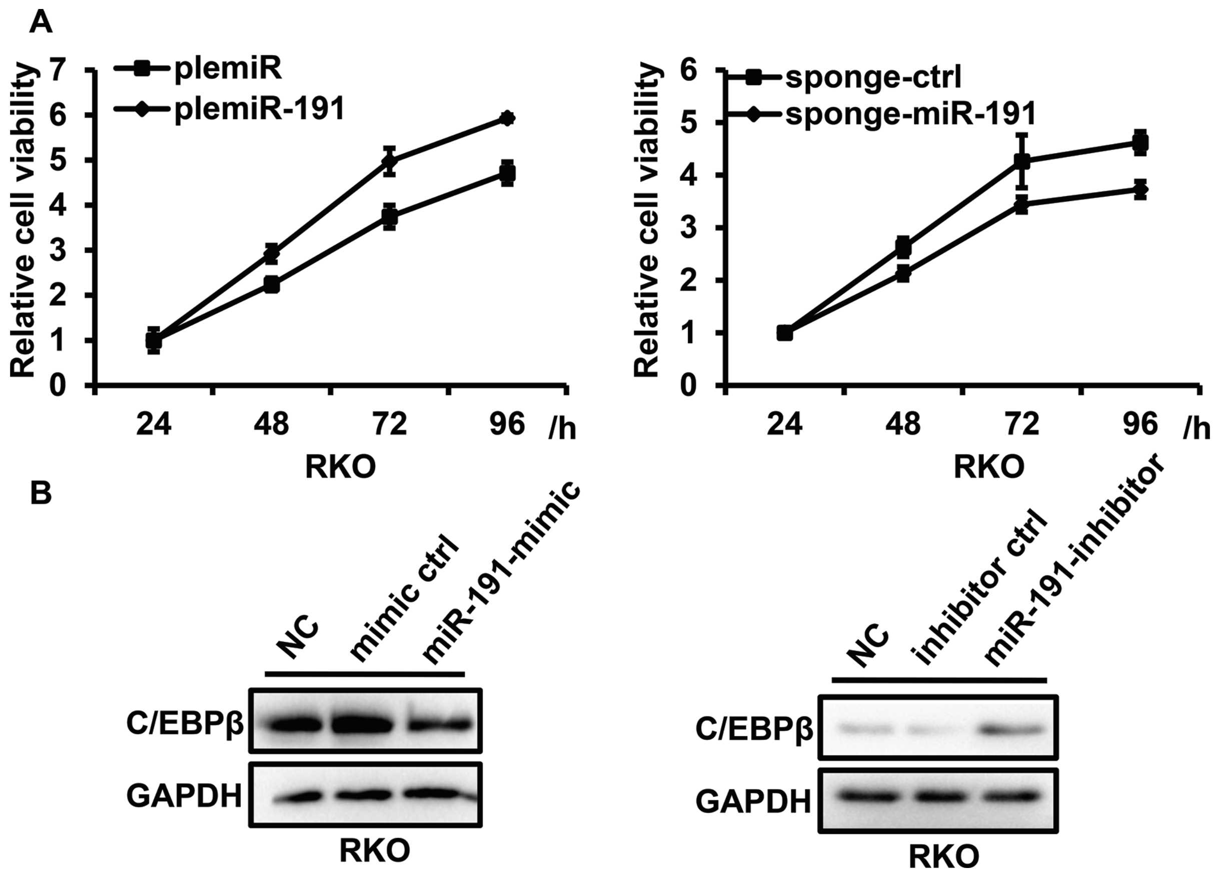


**Supplementary Figure 1: miR-191 increased cell viability and mediated the down-regulation of C/EBPβ in RKO cell line. (A)** Stable RKO cells transfected with plemiR/plemiR-191 and sponge-ctrl/sponge-miR-191 were generated and CCK8 assays were used to assess the cell viability at the indicated time. **(B)** RKO cells were transfected with mimic ctrl/miR-191-mimic or inhibitor ctrl/miR-191 inhibitor for 48 hours, total protein were extracted and subjected to western blotting. GAPDH served as a loading control. The data represents the means ± SDs.

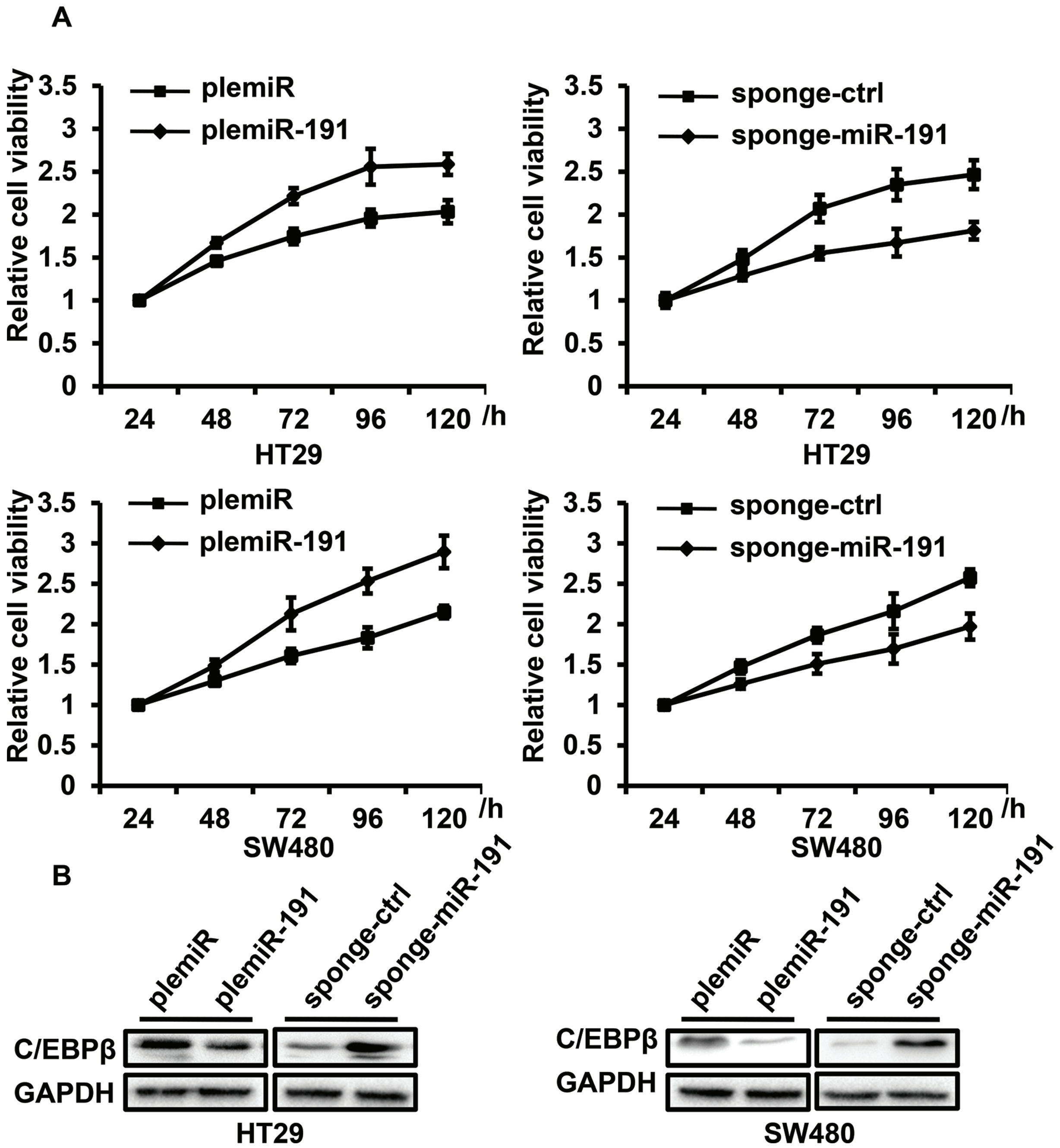


**Supplementary Figure 2: miR-191 induced cell growth and regulated the protein levels of C/EBPβ in HT29 and SW480 cell lines.** Stable HT29 and SW480 cells were generated by infection with lenti-plemiR/plemiR-191 and lenti-sponge-ctrl/sponge-miR-191 viral. **(A)** CCK8 assays were used to assess the cell viability at the indicated time. **(B)** Western blots were performed to detect the protein levels of C/EBPβ in stable HT29 and SW480 cell lines. GAPDH served as a loading control. The data represents the means ± SDs.

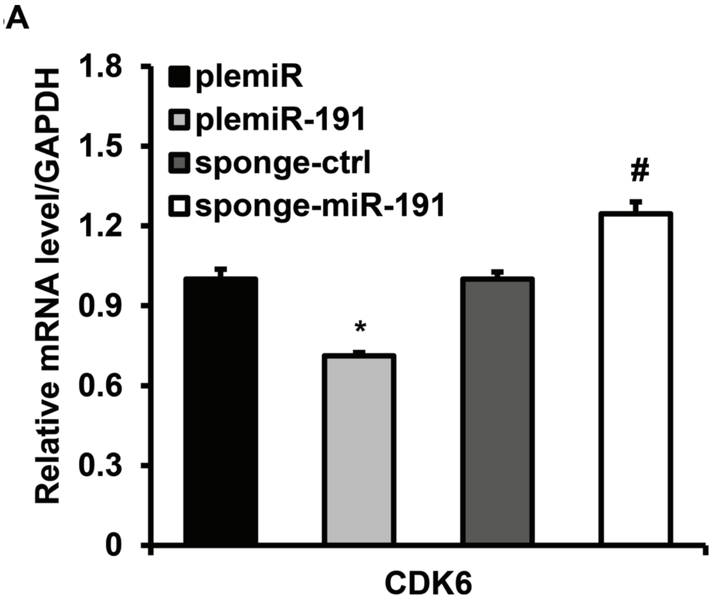


**Supplementary Figure 3: miR-191 decreased the expression of CDK6 in human colorectal cancer HCT116 cells. (A)** Quantitative PCR analysis of the relative expression of CDK6 in HCT116 cells transfected with the indicated plasmids. (*$P < 0.05$ versus plemiR-ctrl and sponge-ctrl) The data represents the means ± SDs.

**Supplementary Table 1: The clinical characteristics of the colorectal cancer and adjacent non-cancer tissues used in our study**

| NO. | Sex | Age | Case classification | TNM stages | Clinical stages | Histologic Grade |
|---|---|---|---|---|---|---|
| 1 | M | 80 | Colon adenoma | T2N0M0 | II | G3 |
| 2 | M | 58 | Rectal adenoma | T1N1M0 | IIIc | G2/G3 |
| 3 | F | 59 | Sigmoid colon adenoma | T1N0M0 | I | G2 |
| 4 | M | 38 | Colon adenoma | T1N0M0 | I | G2 |
| 5 | M | 50 | Rectal adenoma | T4N0M0 | IIIb | G1/G2 |
| 6 | M | 66 | Rectal adenoma | T1N0M0 | I | G2 |
| 7 | F | 73 | Colon mucinous adenoma | T4N0M0 | IIIb | G1 |
| 8 | F | 70 | Rectal tubular adenoma | T1N1M0 | IIIc | G2 |
| 9 | M | 61 | Rectal adenoma | T1N0M0 | I | G2 |
| 10 | F | 69 | Rectal adenoma | T4N0M0 | IIIb | G1 |
| 11 | F | 67 | Sigmoid colon tubular adenoma | T4N1M0 | IIIc | G1 |
| 12 | M | 67 | Colon adenoma | T4N1M0 | IIIc | G3 |
| 13 | M | 75 | Rectal adenoma | T1N1M0 | IIIc | G1/G2 |
| 14 | F | 63 | Rectal tubular adenoma | T4N0M0 | IIIb | G2 |
| 15 | F | 69 | Rectal adenoma | T1N1M0 | IIIc | G2 |
| 16 | M | 56 | Rectal tubular villous adenoma | T4N1M0 | IIIc | G2 |

TNM: Tumor Node Metastasis.
